# Major Gross Lesions of Lung in Cattle Slaughtered at Hawassa Municipal Abattoir, Southern Ethiopia

**DOI:** 10.1155/2017/1702852

**Published:** 2017-12-11

**Authors:** Tesfaheywet Zeryehun, Biruk Alemu

**Affiliations:** ^1^College of Veterinary Medicine, Haramaya University, P.O. Box 301, Haramaya, Ethiopia; ^2^College of Veterinary Medicine, Haramaya University, P.O. Box 138, Haramaya, Ethiopia

## Abstract

A cross-sectional study was conducted from November 2016 to April 2017, to estimate the prevalence of major gross lung lesions in cattle slaughtered at Hawassa Municipal Abattoir, southern Ethiopia. A total of 563 male cattle were examined by antemortem examination, while postmortem lung lesions were done using standard inspection procedures. Association between prevalence and the explanatory variables was estimated by way of chi-square/Fischer's exact tests using statistical packages for social science (SPSS) software. Upon postmortem examination, 96.6% (544/563) of cattle examined had various gross lung lesions. The most important lesions identified were hydatidosis, emphysema, congestion, and atelectasis with prevalence of 45.3%, 19.2%, 18.1%, and 6.4%, respectively. Based on origins of slaughtered animals, hydatid cyst, emphysema, and congestion were common in cattle that came from Tula area with prevalence of 46.3%, 20.4%, and 20%, respectively. The animals from Arsi-negelle and Hawassa were mostly affected by hydatid cyst with prevalence of 44.4% and 42.5%, respectively. Statistically significant association (*p* = .038) was observed between prevalence of atelectasis and body condition of slaughtered cattle. In conclusion, the prevalence of hydatidosis was the major lesion in the lung of slaughtered cattle at Hawassa Municipal Abattoir. Therefore, appropriate disease control strategies should be put in place.

## 1. Introduction

Abattoir plays an important role in screening animal products with various abnormalities and diseases [1, 2]. The aim of meat inspection in abattoir is to provide information that can be utilized for animal diseases control in addition to public health protection and providing risk free products to the society [3]. In Ethiopian abattoirs, the major factors responsible for low productivity in livestock are parasitic diseases. One of the main parasitic causes of lung lesion during postmortem inspection is hydatid cyst (Hydatidosis). Hydatidosis (hydatid disease) is the disease caused by metacestode stage of* Echinococcus* genus. It has been known and documented in Ethiopia as early as the 1970s [4–8].

Several reports had indicated that hydatidosis is widely prevalent in livestock population of various regions of Ethiopia [9, 10]. Hydatidosis is characterized by cyst containing numerous tiny protoscolices that most often develop in the liver and lungs and also develop in the kidneys, spleen, and heart [10, 11]. The pathogenicity of hydatidosis heavily depends on the extent and severity of infection and the organ on which it is situated. The occasional rupture of hydatid cysts often leads to sudden death due to anaphylaxis, haemorrhage, and metastasis [12]. Calcified cysts have a gritty sound upon incision with knife and when observed grossly the cyst is white or grey and irregularly rounded and frequently honey combed. Hydatid cyst contains semisolid material on which there may be deposition of calcium salts to form calcified cyst. Hydatidosis caused by the metacestode of* Echinococcus granulosus* is a widely spread parasitic zoonosis that had caused public health problems in many countries [13, 14].

In addition to hydatidosis, suffocation of animals due to overcrowding in the lairages, lack of enough rest before slaughter, and exposure to bacterial and/or viral infections may lead to development of emphysema and pneumonia [15, 16]. Pneumonia is an inflammation of the lung characterized by enlargement, hyperaemia, and sometimes oedema and most commonly caused by infectious or noninfectious agents. Atelectasis is also another lesion of the lung as a result of collapse of the alveoli due to failure of the alveoli to inflate or because of compression of the alveoli while emphysema occurs due to destruction of connective tissue of the lung, including the supporting and elastic tissue of pulmonary parenchyma. It also occurs in an abnormal permanent accumulation of air in the lungs associated with some disease conditions and is caused by an obstruction to the outflow of air or by extensive gasping respiration during slaughter procedures. The emphysema lesions on lung can be examined grossly by appreciating pale, enlarged greyish-yellow, pearl like shiny, puffy, and crepitant feel upon observation and palpation of the lung [14, 17].

In Ethiopia, meat inspection was started in the 1910s with the aim of improving productivity and trade in animals and animal products as well as protecting the public from zoonotic diseases [18]. Meat inspection is known to provide valuable information on prevalence of disease of public health and economic importance [19]. Studies conducted in various abattoirs in Ethiopia have revealed that lung lesions are among the important reasons for condemnation of organs every year [20, 21]. Lung lesions are known to unveil several clues about diseases of public health importance such as tuberculosis, hydatidosis, and cysticercosis [15]. Hence examination of lungs much closer could bring out valuable data for development of disease prevention strategies and/or programs. Several studies that have been conducted in most abattoirs in Ethiopia focused on major causes of organ condemnation [9, 20–23]. But closer examination of lung lesions and their prevalence has not been adequately addressed although closely examining the gross lesions in the lung could provide adequate information to pinpoint specific lesions which might have public health and economic importance. Therefore, the objective of the current study was to estimate the prevalence of major gross lesions on lungs of cattle slaughtered in Hawassa Municipal Abattoir.

## 2. Materials and Methods

### 2.1. Study Area Description

**Figure 1:**
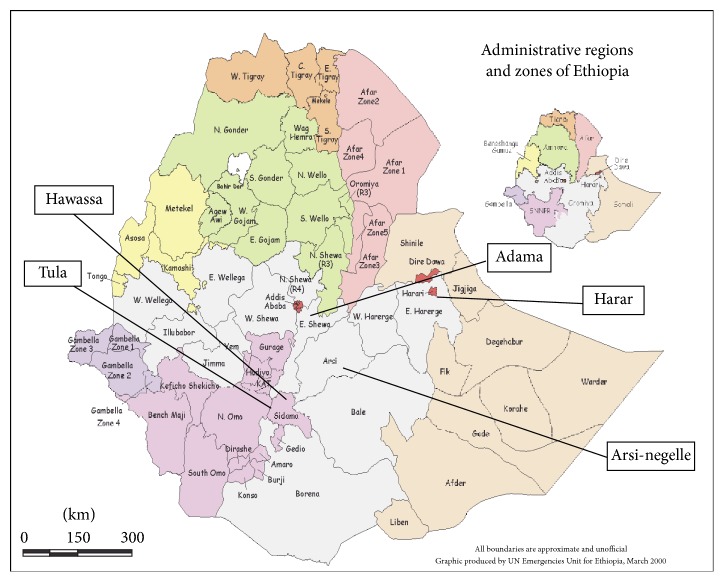
Map of Ethiopia, showing the probable origins of the slaughtered cattle [24].

The study was conducted in Hawassa Municipal Abattoir which is found in southern nations nationalities and people's region (SNNPR), 275 km south of Addis Ababa. As can be seen in Figure 1 the geographical location of this site is between 4°27′ and 8°30′ latitude north and 34°21′ and 39°1′ longitude east. The annual rainfall and temperature of the area vary within 800–1000 mm and 20.1–25°C, respectively [24].

### 2.2. Hawassa Municipal Abattoir

It is administered by the town municipality and provides slaughter and inspection services for three butcher shops in the town. On average, 50 cattle and 30 sheep and goats are slaughtered each day. The overall abattoir environment falls short of the standard level. It is operated by one junior veterinarian and three assistant meat inspectors.

### 2.3. Study Population

A total of 563 male cattle destined to be slaughtered at Hawassa Municipal Abattoir were inspected during antemortem and postmortem inspections. Each of study animals was given an identification number on its body with a color marker during antemortem inspection.

### 2.4. Study Design and Study Methodology

The cross-sectional study was conducted on cattle slaughtered in the Hawassa Municipal Abattoir and during the period from November to April 2017. Three visits per week were made randomly out of five slaughter days in a week. In each visit, antemortem examination and postmortem examination were conducted. 

*Antemortem Examination*. Antemortem inspection was conducted on individual animals, while the animals were allowed to enter into the lairage according to the standard of antemortem examination procedures given by Gracey et al. [3]. Upon the regular visits in antemortem examination, factors such as breed, sex, age, origin of animals, and body condition of each animal were recorded. Estimation of age was carried out by dentition method based on procedures described by Gatenby [25]; accordingly cattle were grouped into young adult (3 to 6 years), adult (6–8 years), and old (>8 years), while the body condition score was grouped as poor, medium, and good on the basis of body condition scoring guideline of zebu cattle [26]. 

*Postmortem Examination*. During postmortem, lung examination was conducted by visualization, palpation, and incisions, where necessary, for the presence of cyst or parasites and other gross abnormalities. Pathological lesions were differentiated according to guidelines on meat inspection for developing countries [19].

### 2.5. Sample Size Determination

Since there is no similar research done in the area, expected prevalence of 50% is assumed based on the rule of thumb. The sample size for the study was calculated by using formula given by Thrufield [27] with 95% confidence level and required 5% precision as shown below:

(1) $$\begin{array}{c} N=\frac{{\left(1.96\right)}^{2}P_{exp}\left(1-P_{exp}\right)}{{\left(d\right)}^{2}}, \end{array}$$

where *N* is required sample size, *P*_exp_ is expected prevalence, and *d* is required precision.

By using the above formula, the required sample size was calculated to be 384, but 179 animals were additionally sampled to increase the precision of the estimated prevalence of the lung lesions, thereby making the total sample size 563.

### 2.6. Data Analysis

Data obtained from antemortem and postmortem findings were entered into Microsoft Excel 2007 computer program. The association and effects of different explanatory variables (breed, age, and body condition score) on the prevalence and distribution of lesion were analyzed using chi-square/Fischer's exact test and binary logistic regression method. A statistically significant association among variables was considered to exist if *p* value was less than 0.05 (*p* < 0.05) at 95% confidence level. Then data were analyzed using SPSS for windows version 20.0 (SPSS Inc., Chicago, Illinois, USA) software.

## 3. Result and Discussion

### 3.1. Results

A total of 563 cattle were slaughtered and examined during the abattoir visit. Out of these animals, the lungs of 544 (96.6%) had various gross lesions during postmortem examination. As shown in Table 1, out of the total examined animals, 96.8% of the local breed and 92.3% of the cross breed were affected by lung lesions. The most prevalent lesions encountered in the lung in each local and cross breed were hydatid cyst (45.6%, 38.5%), emphysema (19.6%, 11.5%), and congestion (18.2%, 15.4%), in (local, exotic) breeds, respectively. The commonest lesion in the lung of both breeds was hydatid cyst and sometimes the hydatid cyst may be found to be associated with emphysema. Congested lung was also commonly seen in the slaughtered animals in both breeds. Although infrequent, lesions such as atelectasis, pneumonia, and consolidation of lung were also recorded.

Assessments of the lung lesions with regard to the various age groups of the slaughtered animals were made as shown in Table 2. The frequencies of the lesions were higher on young cattle followed by adult and old cattle. Nonetheless, there was no significance difference (*p* < 0.05) between the prevalence of each lesion and age group of cattle.

Table 3 shows the distributions of lung lesions based on body condition score of the slaughtered animals. In this regard, it was found out that cattle with medium body condition score had higher percentage of lung lesions (99.7%) compared to cattle with poor (88.4%) and good (77%) body condition score. It was observed that atelectasis was highly prevalent in cattle with poor (11.5%) and medium (7.4%) body condition score compared to those with good (3.4%) body condition scores, and the association was statistically significant (*p* = .038).

**Figure 2:**
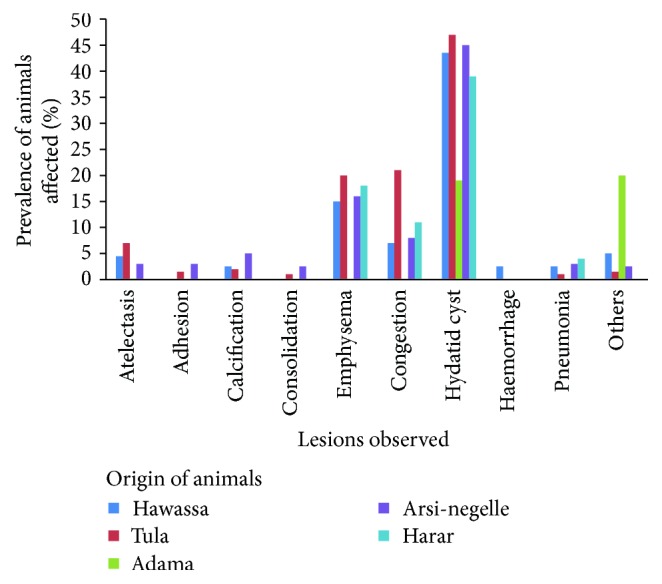
Prevalence of distribution of lesions based on the origin of slaughtered animals.

The major gross lung lesions encountered based on the suspected origins of slaughtered animals in the present study were shown in Figure 2. Hydatid cyst, emphysema, congestion, and atelectasis were common in Tula with prevalence of 46.3%, 20.4%, 20%, and 7.2%, respectively. The animals from Arsi-negelle and Hawassa were mostly affected by hydatid cyst with prevalence of 44.4% and 42.5%, respectively.

### 3.2. Discussion

The present study revealed a very high overall prevalence of lung lesions (96.6%) (544/563) in slaughtered cattle examined in Hawassa Municipal Abattoir. The finding of higher prevalence in the current study affirms that lungs are prone to exposure to physical, chemical, and biological injuries owing to their anatomical and histological characteristics [17]. The prevalence of pulmonary lesion in the present study is very high compared with previous studies. For example, Fekadu et al. [28] and Nebyuu et al. [29] reported 46.22% from Jimma Municipal Abattoir and 15.5% from Nekemit Municipal Abattoir, respectively. This higher prevalence of lung lesion in the present study area might be attributed to the poor prevention of diseases in the area.

The most commonly encountered pulmonary lesions in the current study were hydatid cyst (45.3%), emphysema (19.2%), and congestion (18.1%) in a decreasing order. The hydatid cyst prevalence in the lung (45.3%) in the current study is higher than the hydatid lesions reported by Mellau et al. [14], Asmare et al. [30], and Fekadu et al. [28] who reported 22.2%, 35.7%, and 35.85%, respectively. On the contrary, the hydatid cyst prevalence was lower than the findings of Abebe et al. [31] who reported a higher prevalence of 65.5% from Gondar Abattoir. The home-kept dogs and cats that feed on uncooked lungs, attitude of people to pet animals, culture of the community, and home slaughtering which is commonly used in our country [32] might have contributed to the higher occurrence of hydatid lesion in the present study area.

The frequency of the hydatid cyst lesion on the local breed cattle examined in the current study is comparably more (18.2%) than the frequency of the lesion in the cross breeds (15.4%). This may be because of higher prevalence of hydatidosis in the local breed that has important role in the formation of emphysema in the lung of cattle.

The high prevalence of hydatid lesion (51.9%) in old, (43.5%) in adult, and (38.1%) in young cattle strengthens the higher prevalence of cystic echinococcosis in adult than young cattle reported in Ethiopia by Abebe et al. [31] who registered prevalence of 22.4% in adult and 15.7% in young. Old cattle have greater chance of exposure to more number of infective stages due to longer duration of time than adult and young cattle [31, 32].

The frequency of emphysematous lung lesion in the present study was 18.1% which was higher than the findings of Fasil [33] and Fekadu et al. [28] who reported 1.2% and 6.77%, respectively, but slightly similar to Abayneh (1999) who reported 16.53% in cattle slaughtered at Assela Municipal Abattoir. This might indicate the poor control measures practiced in the study area. However, the report from Addis Ababa Municipal Abattoir by Seboka [34] and the report from Tanzania by Kambarage et al. [35] showed higher prevalence of 43.75% and 22%, respectively. The discrepancy with the present study may be due to agroecology of the area in which some diseases are endemic to specific agroecology where the causative agent or its intermediate host may find favorable conditions. According to FAO [36], cattle have well developed interlobular septa and lack collateral ventilation, making them more susceptible to interstitial emphysema.

Atelectasis was the highest among the least reported pulmonary lesions. This lung lesion might be the result of exposure of animals to stress factors like dust and overcrowding and exhaustion from long treks in search of pasture and water during the dry season may also contribute to respiratory conditions [21]. The current study showed that there was significant association (*p* = 0.038) in the prevalence of bovine lung atelectasis among animals with different body conditions, that is, higher in animals having poor body condition (11.5%) next to animals having medium body condition (7.4%) but lowest in animals having good body condition (3.4%). Animals with poor body condition are most likely affected by a multitude of diseases that affect the lung to the extent of causing severe collapse of lung tissue. For example, the highest prevalence of hydatidosis in the present study could be among the causes of atelectasis.

The prevalence of pneumonia and that of consolidation lesions also were 0.9% each in the current study. The prevalence of pneumonia in the present study agrees with those reported by Fekadu et al. [28] with 1.11% and strongly disagrees with the higher prevalence reported by Cadmus and Adesokan [15] on cattle slaughtered in Nigeria, Ahmed et al. [37] in Egypt, and Kambarage et al. [35] on slaughtered cattle in Tanzania, who reported prevalence of pneumonia to be 31.02%, 28.7%, and 3.33%, respectively. Pneumonia may affect animals that are transported on foot to the abattoir because of transportation stress and starvation in addition to endemic disease such as pasteurellosis and animals having traumatically penetrated lung. Suffocation of animals due to overcrowding in the lairages, lack of enough rest before slaughter, and exposure to bacterial and/or viral infections may lead to development of emphysema and pneumonia [15, 16].

Based on the distribution of pulmonary lesion with regard to site of origin of slaughtered animals, hydatid cyst lesions are more prevalent in lung of cattle slaughtered from Tula, Arsi-negelle, and Hawassa than other origins with prevalence of 46.3%, 44.4%, and 42.5%, respectively. However, the origin of animals has no significant association (*p* > 0.05) with the prevalence of hydatidosis. The discrepancies in the prevalence of hydatidosis with regard to origin of slaughtered animals might be attributed to the variations in the awareness of cattle owners, differences in agroecological conditions, and livestock management system. Furthermore, prevalence of diseases at the different origin sites could be affected by the rate of transmission of echinococcosis/hydatidosis [17]. The home-kept dogs and cats that feed on uncooked lungs, attitude of people to pet animals, culture of the community, and home slaughtering which is commonly used in our country also have a great role in the wide spread of cystic echinococcosis [32, 38, 39].

## 4. Conclusions

The result of the current study identified the major gross lung lesions in slaughtered cattle in the area and their prevalence. The study concluded that hydatidosis, emphysema, and congestion were the common gross lesions encountered in the Hawassa Municipal Abattoir mostly in the animals that came from Tula and Hawassa. Therefore, the Hawassa Municipal Abattoir should have to facilitate proper disposal way of abattoir leftovers such as preparing room or burring spaces for the condemned lung and other organs. Good meat inspection techniques should be practiced in the abattoirs. Awareness creation workshops and campaigns should be organized by municipal abattoirs to improve animal owner's attitude in keeping animals away from sources of infection coupled with better livestock management system. Furthermore, it is important to give special attention to identifying the risk factors and causative agents of lung lesions by conducting further studies in order to put appropriate control measures in place.

## Figures and Tables

**Table 1:** Percentage of distribution of gross lung lesions in the breed categories.

Breed						
Lesions	Total number of lesions	Local (*n* = 537)	CI^*∗∗*^ (*L*–*U*%)	Cross (*n* = 26)	CI (*L*–*U*%)	*p* value
Atelectasis	36 (6.4%)	34 (6.3%)	4.42–8.74	2 (7.7%)	0.95–25.13	.679
Adhesion	8 (1.4%)	7 (1.3%)	0.53–2.67	1 (3.8%)	0.10–19.64	.317
Calcification	13 (2.3%)	11 (2.0%)	1.03–3.64	2 (7.7%)	0.95–25.13	.117
Consolidation	5 (.9%)	4 (.7%)	0.20–1.90	1 (3.8%)	0.10–19.64	.211
Emphysema	108 (19.2%)	105 (19.6%)	16.28–23.16	3 (11.5%)	2.45–30.15	.445
Congestion	102 (18.1%)	98 (18.2%)	15.07–21.78	4 (15.4%)	4.36–34.87	1.000
Hydatid cyst	255 (45.3%)	245 (45.6%)	41.35–49.94	10 (38.5%)	20.23–59.43	.548
Haemorrhage	2 (.4%)	2 (.4%)	0.05–1.34	0 (.0%)	0.00–13.23	1.000
Pneumonia	5 (.9%)	5 (.9%)	0.30–2.16	0 (.0%)	0.00–13.23	1.000
Others^*∗*^	10 (1.8%)	9 (1.7%)	0.77–3.16	1 (3.8%)	0.10–19.64	.379

Total	544 (96.6%)	520 (96.8%)		24 (92.3%)		

^*∗*^Lesions such as fibrosis and abscess; ^*∗∗*^CI—confidence interval; *L*—lower limit; *U*—upper limit.

**Table 2:** Percentage of gross lung lesions in the different age groups.

Age								
Lesions	Total number of lesions^*∗*^	Young (*n* = 21)	CI^*∗∗*^ (*L*–*U*%)	Adult (*n* = 407)	CI (*L*–*U*%)	Old (*n* = 135)	CI (*L*–*U*%)	*p* value
Atelectasis	36 (6.4%)	5 (23.8%)	8.22–47.17	23 (5.7%)	3.62–8.36	8 (5.9%)	2.59–11.34	.129
Adhesion	8 (1.4%)	0	0.00–16.11	5 (1.2%)	0.40–2.84	3 (2.2%)	0.46–6.36	.312
Calcification	13 (2.3%)	0	0.00–16.11	11 (2.7%)	1.36–4.78	2 (1.5%)	0.18–5.25	.715
Consolidation	5 (.9%)	1 (4.8%)	0.12–23.82	4 (1.0%)	0.27–2.50	0	0.00–2.70	.063
Emphysema	108 (19.2%)	5 (23.8%)	8.22–47.17	83 (20.4%)	16.58–24.64	20 (14.8%)	9.29–21.95	.131
Congestion	102 (18.1%)	3 (14.3%)	3.05–36.34	84 (20.6%)	16.81–24.90	15 (11.1%)	6.35–17.66	.052
Hydatid cyst	255 (45.3%)	8 (38.1%)	18.11–61.56	177 (43.5%)	38.61–48.46	70 (51.9%)	43.09–60.53	.071
Haemorrhage	2 (.4%)	0	0.00–16.11	2 (.5%)	0.06–1.76	0	0.00–2.70	-
Pneumonia	5 (.9%)	0	0.00–16.11	3 (.7%)	0.15–2.14	2 (1.5%)	0.18–5.25	.362
Others	10 (1.8%)	0	0.00–16.11	8 (2.0%)	0.85–3.84	2 (1.5%)	0.18–5.25	.987

Total	544 (96.6%)	22 (104.7%)		400 (98.3%)		122 (90.4%)		

^*∗*^In some parts of the results, the percentage of the lesions is greater than one hundred, because there was the opportunity for one lung to be registered more than once if it has got multiple lesions; ^*∗∗*^CI—confidence interval; *L*—lower limit; *U*—upper limit.

**Table 3:** Percentage of gross lung lesions in the different body condition score.

Body condition score								
Lesions	Total number of lesions	Poor (*n* = 26)	CI^*∗∗*^ (*L*–*U*)	Medium (*n* = 363)	CI (*L*–*U*)	Good (*n* = 174)	CI (*L*–*U*)	*p* value
Atelectasis	36 (6.4%)	3 (11.5%)	2.45–30.15	27 (7.4%)	0.96–4.31	6 (3.4%)	1.28–7.35	.038
Adhesion	8 (1.4%)	0	0.00–13.23	6 (1.7%)	0.17–2.40	2 (1.1%)	0.14–4.09	.945
Calcification	13 (2.3%)	1 (3.8%)	0.10–19.64	8 (2.2%)	16.66–25.26	4 (2.3%)	0.63–5.78	.827
Consolidation	5 (.9%)	0	0.00–13.23	3 (.8%)	16.41–24.97	2 (1.1%)	0.14–4.09	.565
Emphysema	108 (19.2%)	4 (15.4%)	4.36–34.87	75 (20.7%)	39.01–49.48	29 (16.7%)	11.46–23.05	.498
Congestion	102 (18.1%)	3 (11.5%)	2.45–30.15	74 (20.4%)	0.01–1.53	25 (14.4%)	9.52–20.48	.325
Hydatid cyst	255(45.3%)	11 (42.3%)	23.35–63.08	160 (44.1%)	0.07–1.98	84 (48.3%)	40.65–55.96	.345
Haemorrhage	2 (.4%)	0	0.00–13.23	1 (.3%)	0.61–3.57	1 (.6%)	0.01–3.16	.530
Pneumonia	5 (.9%)	1 (3.8%)	0.10–19.64	2 (.6%)	0.00–0.00	2 (1.1%)	0.14–4.09	.792
Others^*∗*^	10 (1.8%)	0	0.00–13.23	6 (1.7%)	0.00–0.00	4 (2.3%)	0.63–5.78	.414

Total	544(96.6%)	23 (88.4%)		362 (99.7%)		159 (91.3%)		

^*∗*^Lesions such as fibrosis and abscess; ^*∗∗*^CI—confidence interval; *L*—lower limit; *U*—upper limit.
